# Unveiling the Antimalarial Potential of Leaf Extracts of *Mussaenda erythrophylla* Schum. & Thonn. and *Mussaenda philippica Dona Luz* x *M. flava* in Mice

**DOI:** 10.1155/jotm/4178099

**Published:** 2026-02-24

**Authors:** Prapaporn Chaniad, Arisara Phuwajaroanpong, Walaiporn Plirat, Atthaphon Konyanee, Parnpen Viriyavejakul, Abdi Wira Septama, Chuchard Punsawad

**Affiliations:** ^1^ Department of Medical Sciences, School of Medicine, Walailak University, Nakhon Si Thammarat, 80160, Thailand, wu.ac.th; ^2^ Center of Excellence in Tropical Pathobiology, Walailak University, Nakhon Si Thammarat, 80160, Thailand, wu.ac.th; ^3^ School of Allied Health Sciences, Walailak University, Nakhon Si Thammarat, 80160, Thailand, wu.ac.th; ^4^ Department of Tropical Pathology, Faculty of Tropical Medicine, Mahidol University, Bangkok, 10400, Thailand, mahidol.ac.th; ^5^ Research Center for Pharmaceutical Ingredient and Traditional Medicine, National Research and Innovation Agency (BRIN), Cibinong Science Center, Cibinong, Bogor, 16915, Indonesia, brin.go.id

**Keywords:** antimalarial activity, malaria, *Mussaenda erythrophylla*, *Mussaenda philippica*, *Plasmodium berghei*

## Abstract

Malaria remains a major global public health concern, particularly in tropical regions. The increasing resistance to the current antimalarial drugs highlights the urgent need for new and effective therapies. Medicinal plants offer a promising source of novel and affordable antimalarial compounds for drug development. This study aimed to evaluate the antimalarial potential and acute oral toxicity of ethanolic leaf extract of *Mussaenda erythrophylla* Schum. & Thonn. (*M. erythrophylla* or Dona Trining) and *Mussaenda philippica Dona Luz* x *M. flava* (*M. philippica* or Dona Marmalade). Male Institute of Cancer Research (ICR) mice were treated with crude extracts at doses of 200, 400, and 600 mg/kg body weight, and the antimalarial activity was assessed using a 4‐day suppressive test against *Plasmodium berghei* ANKA strain. The ethanolic leaf extract of *M. erythrophylla* exhibited a parasite suppression of 12.31%, 39.59%, and 59.76% at 200, 400, and 600 mg/kg, respectively. Similarly, *M. philippica* leaf extract suppressed parasitemia by 36.18%, 36.40%, and 71.02% at the corresponding doses. All extract concentrations, except for the 200 mg/kg dose of *M. erythrophylla*, exhibited higher effects compared to the negative controls (*p* < 0.05). At a dose of 2000 mg/kg, acute oral toxicity testing revealed no changes in ALT, ALP, BUN, or creatinine levels compared to controls, although AST levels were elevated. This increase was considered a possible mild adaptive response rather than a sign of overt toxicity. No alterations were observed in the physical activity or behavior of the mice, including piloerection, lacrimation, feeding activity, abnormal secretions, sleep patterns, or unusual excitement. Additionally, neither crude extract induced histological alterations in liver or kidney tissues. In conclusion, ethanolic leaf extracts of *M. erythrophylla* and *M. philippica* demonstrated promising antimalarial activity and were deemed safe at the tested doses, demonstrating safety up to 2000 mg/kg with only mild AST elevation and no observable histopathological damage. Extending the previous in vitro results of potent antimalarial activity and low cytotoxicity, our in vivo findings strongly support the efficacy and safety of these extracts. Further research is recommended to isolate and identify the active compounds responsible for the observed effects.

## 1. Introduction

Malaria is a deadly parasitic infectious illness in the tropical and subtropical areas of Asia and Africa [1, 2]. By 2023, there will be an estimated 263 million malaria cases worldwide, resulting in approximately 597,000 deaths. Although the African region bears the majority of the burden, the Southeast Asian region reports approximately four million cases (1.5% of global cases) annually. Notably, Myanmar and Thailand will experience substantial increases in malaria incidence between 2022 and 2023. In Myanmar, reported cases have risen over tenfold, from 78,000 in 2019 to 847,000 in 2023, whereas in Thailand, indigenous cases have more than tripled, from 2426 in 2021 to 9169 in 2023 [3]. Historically, malaria has been treated with quinine, derived from Cinchona bark, followed by chloroquine (CQ) and sulfadoxine–pyrimethamine (SP), as first‐line therapies. Later, mefloquine was introduced; however, the emergence of parasitic resistance has limited the long‐term effectiveness of these drugs [4]. Currently, artemisinin‐based combination therapies (ACTs) are the recommended first‐line treatment, while severe malaria cases are managed with injectable artesunate or quinine depending on the clinical guidelines [5, 6]. Currently, the most severe challenge in the fight against malaria is parasitic resistance to antimalarial medications [7]. In Southeast Asia, 10% of the first‐line treatments fail to cure *Plasmodium falciparum* infections, whereas 93% fail in Thailand [8]. Therefore, there have been constant efforts to search for alternative antimalarial agents that are effective against parasitic resistance [3].

Traditional medicinal plants are widely used in developing countries, with 80% of the population relying on them for treatment of various illnesses, including malaria [9, 10]. Several clinically important antimalarial agents such as artemisinin and quinine are plant‐derived compounds [11, 12]. Medicinal plants contain numerous bioactive compounds, including secondary metabolites, which can serve as potential antimalarial agents [13]. Consequently, they are key sources for novel antimalarial therapies.

*M. erythrophylla* is a sprawling shrub with red sepals and white flowers with red centers. It grows in deciduous forests and woodlands, which are distributed throughout tropical Africa and found in most tropical countries. *M. erythrophylla* is used in traditional African and Asian medicine to treat eye infections, intestinal parasites, body aches, diarrhea, and dysentery. In addition, this plant has been reported in some pharmacological studies for its anti‐inflammatory and antipyretic activities, which are used to treat inflammation, fever, and common symptoms of malaria [14]. Coumarins, flavonoid glucosides, quinine acid derivatives, triterpenoids, monoglycerides, steroids, tetraterpenoids, and polyols are found in the extracts of the aerial parts of *M. erythrophylla* extract [15]. These classes of compounds, including triterpenoids and flavonoids, have widely been reported to exhibit antimalarial or immunomodulatory properties in related species, suggesting their potential relevance to antiplasmodial activity [16].

*M. philippica* is a shrub with hardy and vigorous variable ovate to lanceolate leaves and inflorescence‐enlarged flowers. The flowers are yellow or yellow‐orange to orange in color and have orange sepals [17, 18]. *M. philippica’*s varied components are effective against various disorders; the bark is used to treat stomach aches, diarrhea, lung, and chest infections, and fully mature sepals for jaundice [19]. Phytoconstituents isolated from *M. philippica* include iridoid glycosides and flavones [20]. Iridoid glycosides are known in other medicinal plants for antimicrobial, anti‐inflammatory, and antiprotozoal activities, providing a plausible mechanistic basis for antimalarial potential [21–23]. Triterpenoids, the major constituents of the leaves and roots of *M. parviflora*, are useful in the treatment of malarial fever [20]. Compared to many other genera in the Rubiaceae family, Mussaenda is distinguished by its promising antimalarial activity observed in *in vitro* screenings, its diverse yet relatively underexplored phytochemical profile, and its documented ethnobotanical uses, including the treatment of fever, diarrhea, and infectious diseases. Several studies have used medicinal plants as potential sources of novel antimalarial agents [24]. The Rubiaceae family has long been associated with antimalarial activity, most notably through quinine isolated from Cinchona trees (Rubiaceae) [25, 26].

In our previous studies, we screened the antimalarial activities of aqueous and ethanolic extracts against a CQ‐resistant *Plasmodium falciparum* (K1) strain using a lactate dehydrogenase (*p*LDH) assay. The results demonstrated that the ethanolic extracts of the leaves of *M. erythrophylla* and *M. philippica* exhibited superior antimalarial activity compared to the aqueous extracts. Specifically, the IC_50_ value of the ethanolic extract of *M. erythrophylla* was 3.7 μg/mL, whereas the aqueous extract had an IC_50_ of 222.1 μg/mL. Similarly, for *M. philippica*, the ethanolic extract (IC_50_ = 5.9 μg/mL) showed better antimalarial activity than the aqueous extract (IC_50_ = 52.2 μg/mL). These data indicate potent in vitro antimalarial activity and provide a strong rationale for selecting ethanolic extracts for subsequent in vivo evaluation. Additionally, the ethanolic extracts demonstrated low cytotoxicity to Vero cells (CC_50_ values of 114.7 μg/mL for *M. erythrophylla* and 150.7 μg/mL for *M. philippica*), supporting their suitability for in vivo studies. The selectivity index (SI), which estimates the potential of the extracts to inhibit parasite growth without causing toxicity, was 30.7 for *M. erythrophylla* and 25.7 for *M. philippica* [27]. These results suggested that both extracts possessed significant antimalarial activity with low cytotoxicity and favorable selectivity. Based on these preliminary findings, the ethanolic extracts were selected for in vivo antimalarial studies.

Our previous studies demonstrated the potent in vitro antimalarial activity of ethanolic extracts, along with low cytotoxicity and favorable selectivity indices. However, these findings are inherently limited by the constraints of in vitro systems and may not fully predict in vivo efficacy. This is because such models lack host immune interactions, metabolic transformations, and pharmacokinetic variability, all of which can influence therapeutic outcomes [4]. Therefore, in vivo evaluation is necessary to confirm the biological relevance, assess systemic effects, and determine the actual extract.

Notably, the in vivo antimalarial efficacy of these species has not yet been investigated. Collectively, these considerations supported the selection of *M. erythrophylla* and *M. philippica* as novel candidates for further in vivo antimalarial evaluation.

## 2. Materials and Methods

### 2.1. Preparation of Plant Samples

Fresh leaves of *M. erythrophylla* and *M. philippica* were harvested from Khuan Khanun District, Phatthalung Province, Thailand, at coordinates 7°44′6″N, 100°0′36″E and 7°44′5.7″N, 100°0′36″E, respectively. Authentication of the plant materials was conducted by Assoc. Prof. Tanomjit Supavita, an expert botanist who examined the detailed morphological characteristics. Identification was performed following authoritative taxonomic references to ensure accurate species determination [28, 29]. All samples were collected during the same season and from the same geographical location to minimize phytochemical variability. Voucher specimens of *M. erythrophylla* (SMD233048005) and *M. philippica* (SMD233048016) were obtained from the School of Medicine, Walailak University. Authorization for the collection of plant materials was obtained in accordance with the relevant guidelines and regulations set by the Plant Varieties Protection Department of Agriculture, Ministry of Agriculture and Cooperatives, Thailand.

### 2.2. Extraction of Plant Materials

A previous in vitro study found that the ethanolic extracts of *M. erythrophylla* and *M. philippica* exhibit strong antimalarial activity. Therefore, 95% ethanol was used as the solvent for extraction via maceration under controlled conditions, including the solvent‐to‐sample ratio, maceration temperature, extraction duration, and drying temperature. Fresh leaves of *M. erythrophylla* and *M. philippica* were washed with distilled water and then dried in a hot air oven (Memmert, Model SFE600, Schwabach, Germany) at a consistent temperature of 50°C for 5 days. The leaves were then pulverized using a grinder (Taizhou Jincheng Pharmaceutical Machinery Co., Ltd., Model SF, Jiangsu, China). Leaf powder (200 g) was macerated in 2 L of ethanol at 25°C for 3 days. Extracts were filtered (Whatman No. 1), re‐extracted twice, combined, concentrated at 50°C under reduced pressure (Rotavapor, Buchi), and freeze‐dried (Christ Gamma 2–16). Dried extracts were weighed for yield and stored at 4°C in airtight containers to preserve stability [27]. The extraction yield (%) was determined as follows: extraction yield (%) = [(weight of crude extract)/(weight of dried plant sample)] × 100.

The extractive yield from the extraction of the leaves of *M. erythrophylla and M. philippica* in the study using 95% ethanol solution was 17.66 g (8.53%) and 20.16 g (10.08%), respectively (Table 1). The substances obtained after concentrating the extracts were brownish and sticky.

**TABLE 1:** Extraction yield of *M. erythrophylla* and *M. philippica*.

No.	Sample	Extract	Total weight (g)	% yield
1	*M. erythrophylla*	Ethanol	17.66	8.53
2	*M. philippica*	Ethanol	20.16	10.08

### 2.3. Animal Preparation

Male Institute for Cancer Research (ICR) mice (25–30 g, 6–8 weeks) were supplied by Nomura Siam International Co., Ltd., Bangkok, Thailand. Only male ICR mice were used to reduce the variability associated with hormonal fluctuations in females, which can affect metabolic and behavioral responses. The mice were acclimated to the experimental environment for 1 week prior to the study under standard and controlled laboratory conditions. Animal care staff ensured hygiene by cleaning the cages and removing waste daily. The mice were fed pellets and provided with clean drinking water *ad libitum* in a controlled environment. The environment was set at 12‐h light and 12‐h dark cycles, with 22 ± 3°C of room temperature and 50%–60% of humidity. Five animals were housed per cage in transparent plastic cages (220 × 340 × 150 mm) topped with wire lids.

All the experimental procedures were approved by the Animal Ethics Committee of Walailak University (Protocol Number WU‐ACUC‐66008). The research team and animal care staff underwent comprehensive training on the proper handling and use of laboratory animals. The experimental period lasted for approximately 30 days, during which the animals were observed twice daily to monitor their welfare and behavior. This study strictly followed all relevant guidelines and regulations for animal use, ensuring compliance with the Animal Research: Reporting of In Vivo Experiments (ARRIVE) Guidelines. Surgical procedures were performed under 2% isoflurane anesthesia (Piramal Critical Care, USA) with oxygen as the carrier gas in an induction chamber, with diligent efforts to reduce animal distress. Animals with severe symptoms, such as the inability to move, unresponsiveness to external stimuli, or coma, were immediately and humanely euthanized using 2% isoflurane anesthesia, followed by cardiac puncture to minimize pain and distress.

### 2.4. Malaria Parasite Preparation

The rodent malarial parasite *Plasmodium berghei* strain ANKA was obtained from BEI Resources (NIAID, National Institutes of Health, Bethesda, MD, USA). To initiate the inoculation process, 0.2 mL of infected blood cells was injected intraperitoneally (IP) into donor mice.

Blood was collected from donor mice by cardiac puncture when parasitemia reached 20%–30% and was transferred into heparinized tubes. The number of parasitized erythrocytes was determined using a hemocytometer, and the blood was subsequently diluted with sterile PBS to achieve a standardized inoculum of 1 × 10^7^ infected red blood cells in 200 μL.

### 2.5. Assessment of Antimalarial Activity

#### 2.5.1. Experimental Design

A 4‐day suppressive test (Peter’s test) against the CQ‐sensitive strain of *P. berghei* ANKA was performed to assess the in vivo antimalarial activity of the *M. erythrophylla* and *M. philippica* extracts [30, 31]. This test was conducted to assess the schizonticidal activity of the extracts against *P. berghei* in mice during early stage infection [32]. Forty‐five mice were randomly allocated into nine groups, each consisting of five animals, to minimize the selection bias. However, the treatment administration and outcome assessments were not blinded owing to the practical constraints of handling and dosing the plant extracts, which required direct observation and manual administration. Standardized protocols were applied for dosing and outcome measurements to minimize potential bias and ensure reproducibility of the results.

The nine groups included a negative control group and two positive controls that received either artesunate or CQ. Based on previous studies, six treatment groups were administered *M. erythrophylla* or *M. philippica* extracts at 200, 400, or 600 mg/kg, corresponding to low, moderate, and high doses, respectively [31, 33]. Artesunate was included because of its rapid action and relevance as a first‐line antimalarial drug representing the current clinical therapy, whereas CQ served as a classical standard for *P. berghei*‐sensitive strains. Detailed information on the allocation of mice and corresponding treatments is provided in Table 2.

**TABLE 2:** Grouping of mice in the 4 day suppressive test.

Group number (*n* = 5/group)	Treatment (dose)
1. Negative control	7% Tween 80 and 3% ethanol in distilled water
2. Positive Control‐I	Artesunate (6 mg/kg)
3. Positive Control‐II	Chloroquine (25 mg/kg)
4. Testing Group 4	*M. erythrophylla* extract at 200 mg/kg
5. Testing Group 5	*M. erythrophylla* extract at 400 mg/kg
6. Testing Group 6	*M. erythrophylla* extract at 600 mg/kg
7. Testing Group 7	*M. philippica* extract at 200 mg/kg
8. Testing Group 8	*M. philippica* extract at 400 mg/kg
9. Testing Group 9	*M. philippica* extract at 600 mg/kg

#### 2.5.2. Infection Procedure

To initiate infection, the mice were administered an intraperitoneal injection of 200 μL containing 1 × 10^7^ *P. berghei-*infected red blood cells. The vehicle, drugs, and extracts were administered to the mice at the time interval (3, 24, 48, and 72 h after infection) by oral administration.

#### 2.5.3. Parasitemia Assessment

At 96 h postinfection, following the conclusion of the experiments, all mice were anesthetized with 2% isoflurane inhalation and subsequently euthanized via cardiac puncture. Thin blood film samples were obtained from tail veins. Giemsa solution was used to stain the blood films, which were then examined under a light microscope (Olympus CX31, Tokyo, Japan). The number of infected and noninfected erythrocytes in randomly selected fields during microscopic examination was counted to calculate the proportion of parasitemia. Five fields were counted, and the percentages of parasitemia and inhibition were calculated using the following formulas:

(1) %parasitemia=the number of infected red cellsthe number of total red cells×100,%suppression=mean parasitemia of vehicle group−mean parasitemia of drugs or the extract groupmean parasitemia in vehicle group×100.

### 2.6. Assessment of Acute Oral Toxicity

This test was conducted in accordance with OECD guidelines (Test No. 425) [34]. Twenty male mice were randomly assigned to four groups, each comprising five animals: untreated mice, mice administered 7% Tween 80 and 3% ethanol in distilled water, mice receiving *M. erythrophylla* at 2000 mg/kg, and mice given *M. philippica* at 2000 mg/kg. A dose of 2000 mg/kg was selected for both extracts, as this is the standard limit dose for evaluating substances with anticipated low toxicity. This dose was sufficient to identify potential toxic effects, including mortality, clinical signs, and gross pathological changes, without the need for multiple dose levels.

Before weighing and administering the test substance, the mice were fasted for 3 h. On Day 0, mice were orally administered a single dose of the extract or vehicle via a stomach tube. The treated mice were monitored for physical and behavioral problems within the first 30 min of dosing and then twice daily for 14 days. Changes in skin, hair, eyes, mucous membranes, central nervous system, somatomotor activity, and behavioral patterns were observed. Food and water consumption was documented daily. On Day 15, all the mice were weighed prior to anesthetization with 2% isoflurane and euthanized by cardiac puncture. Blood samples were collected and analyzed for liver and kidney functions using an automated chemistry analyzer (Beckman Coulter, USA). Liver and kidney tissues were histologically examined as described previously [8].

### 2.7. Statistical Analysis

IBM SPSS Statistics (version 17.0; IBM, Armonk, NY, USA) was used for statistical analysis. The mean ± standard deviation (SD) was used to represent all the quantitative data. Normal distribution was assessed using the Kolmogorov–Smirnov test. A one‐way analysis of variance (ANOVA) was performed, followed by Tukey’s post hoc multiple comparison test. A *p* value of less than 0.05 was considered statistically significant for all analyses.

## 3. Results

### 3.1. Four‐Day Suppressive Test

**FIGURE 1:**
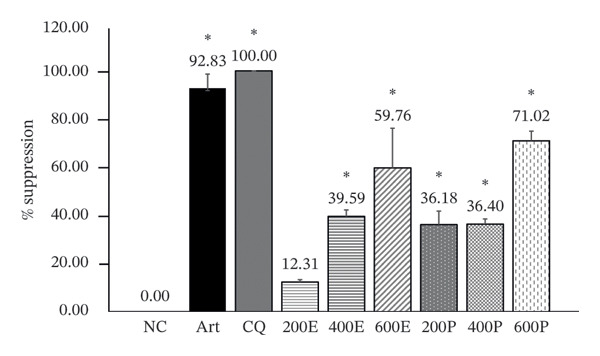
Effects of the ethanolic extracts derived from *M. erythrophylla* and *M. philippica* in mice infected with *P. berghei* ANKA. Results are presented as mean ± standard deviation (SD) (*n* = 5 per group), with a significance level of *p* < 0.05. NC, negative control; Art, artesunate; CQ, chloroquine; 200 E, *M. erythrophylla* extract at 200 mg/kg; 400 E, *M. erythrophylla* extract at 400 mg/kg; 600 E, *M. erythrophylla* extract at 600 mg/kg; 200 P, *M. philippica* extract at 200 mg/kg; 400 P, *M. philippica* extract at 400 mg/kg; 600 P, *M. philippica* extract at 600 mg/kg. ^∗^Compared to NC.

When tested against early infection, the ethanolic leaf extract of *M. erythrophylla* at doses of 200, 400, and 600 mg/kg suppressed parasitemia by 12.31%, 39.59%, and 59.76%, respectively. Moreover, *M. philippica* leaf extract suppressed parasitemia by 36.18%, 36.40%, and 71.02% at the same doses. All extract concentrations exhibited significant suppression of the parasites, except for the 200 mg/kg dose of *M. erythrophylla*, which was notably lower than that of the positive control group and significantly higher than that of the negative control (Figure 1). The antimalarial effects of the ethanolic leaf extracts of *M. erythrophylla* and *M. philippica* were further confirmed based on their half‐maximal effective dose (ED_50_) and 90% effective dose (ED_90_) values (Table 3). *M. erythrophylla* showed an ED_50_ of 524.66 mg/kg and an ED_90_ of 882.74 mg/kg, while *M. philippica* had an ED_50_ of 549.63 mg/kg and an ED_90_ of 711.25 mg/kg. These results indicated that both extracts effectively suppressed parasitemia, with *M. erythrophylla* being slightly more potent at the 50% inhibition level, whereas *M. philippica* required a lower dose to achieve 90% suppression.

**TABLE 3:** ED_50_ and ED_90_ values of *M. erythrophylla* and *M. philippica* extracts against parasitemia.

Extract	ED_50_ (mg/kg)	ED_90_ (mg/kg)
*M. erythrophylla* extract	524.66 ± 67.91	882.74 ± 150.01
*M. philippica* extract	549.63 ± 22.39	711.25 ± 118.99

*Note:* The data are presented as the mean ± SD (*n* = 5 per group).

### 3.2. Acute Oral Toxicity Test

#### 3.2.1. Physical Activity and Behavior Alteration, Food Consumption and Water Uptake, and Body Weight Changes

Mice receiving a single dose of the crude extract exhibited no mortality or observable signs of acute toxicity during the 14‐day observation period. There were no alterations in their physical or behavioral characteristics, including piloerection, lacrimation, feeding activity, abnormal secretions, sleep patterns, or unusual excitement. The average food consumption and water uptake in mice administered a single dose of 2000 mg/kg crude extract, as well as in the negative control group, did not exhibit any statistically significant differences when compared with those in the control group on Days 7 and 14. Additionally, the mean changes in body weight in the group of mice given 2000 mg/kg crude extract and 7% Tween 80 did not show a statistically significant difference compared to those in the control group, as presented in Table 4.

**TABLE 4:** Effects of ethanolic leaf extracts of *M. erythrophylla* and *M. philippica* on body weight, food consumption, and water intake in mice during acute oral toxicity testing.

Group	Food consumption (g)		Water uptake (mL)		Body weight (g)		
	Week 1	Week 2	Week 1	Week 2	D0	D14	% change
Untreated control	27.00 ± 0.96	26.57 ± 1.88	43.42 ± 3.15	40.28 ± 5.23	40.46 ± 2.55	43.22 ± 3.35	6.73 ± 2.03
7% Tween 80	26.14 ± 1.63	25.42 ± 2.91	43.85 ± 4.00	44.28 ± 1.59	40.37 ± 3.08	43.70 ± 3.40	8.26 ± 1.77
*M. erythrophylla*	25.85 ± 1.97	26.00 ± 4.00	48.42 ± 3.26	49.00 ± 2.80	37.62 ± 1.88	39.99 ± 3.40	6.14 ± 3.73
*M. philippica*	27.00 ± 2.49	23.85 ± 1.59	46.14 ± 7.18	45.78 ± 2.62	38.79 ± 1.52	40.75 ± 2.35	4.99 ± 2.30

*Note:* The data are presented as the mean ± SD (*n* = 5 per group), with a significance level of *p* < 0.05.

#### 3.2.2. Biochemical Markers of Liver and Kidney Functions

The biochemical parameters related to liver function in mice receiving a single dose of 2000 mg/kg were not significantly different from those in the control and 7% Tween 80 negative control groups. Although AST levels were slightly higher in mice administered a single dose of 2000 mg/kg extracts than in the untreated control and 7% Tween 80 negative control groups, the values remained within the normal physiological range for mice [35] (Table 5). This indicated that despite the observed increase, there was no clinically relevant liver toxicity. Regarding kidney function biochemical indicators, BUN and creatinine levels in mice administered 2000 mg/kg *M. erythrophylla* and *M. philippica* crude extracts were not significantly different from those in the untreated control and negative control groups, as presented in Table 5.

**TABLE 5:** Effects of the ethanolic extracts of *M. erythrophylla* and *M. philippica* on liver and kidney function in the acute toxicity test.

**Group**	**Liver function test**		
	**AST (U/L)**	**ALT (U/L)**	**ALP (U/L)**

Untreated control	84.60 ± 2.61	51.20 ± 5.12	78.40 ± 8.73
7% Tween 80	56.20 ± 6.94^a^	49.00 ± 4.20	77.20 ± 4.12
*M. erythrophylla* extract	135.20 ± 4.97^a^	52.40 ± 3.21	80.00 ± 1.58
*M. philippica* extract	136.40 ± 12.05^a^	48.20 ± 7.46	78.60 ± 8.17

**Group**	**Kidney function test**		
	**BUN (mg/dL)**	**Creatinine (mg/dL)**	

Untreated control	23.20 ± 11.47	0.33 ± 0.05	
7% Tween 80	21.20 ± 1.48	0.32 ± 0.02	
*M. erythrophylla* extract	23.00 ± 3.54	0.28 ± 0.04	
*M. philippica* extract	20.20 ± 0.84	0.27 ± 0.01	

*Note:* The data are presented as the mean ± SD (*n* = 5 per group), with a significance level of *p* < 0.05.

Abbreviation: AST, aspartate aminotransferase; ALT, alanine aminotransferase; ALP, alkaline phosphatase; BUN, blood urea nitrogen.

^a^In comparison to the untreated control.

#### 3.2.3. Histopathological Examination

Figure 2 shows representative images from the histological examination of the liver and kidney. The liver tissues of mice receiving a single 2000 mg/kg dose exhibited a normal hepatocyte shape characterized by pink cytoplasm, spherical nuclei, and prominent nucleoli. Furthermore, the liver tissues exhibited a normal arrangement of hepatic sinusoids and central veins (Figures 2(c) and 2(d)). Liver tissues exhibited no evidence of sinusoid dilation or inflammatory cell infiltration compared with the control group (Figure 2(a)) and the 7% Tween 80 negative control group (Figure 2(b)). Additionally, kidney sections from the treatment group exhibited normal morphology of the glomerulus, Bowman’s capsule, and renal epithelial cells (Figures 2(g), 2(h)) compared to the control group (Figure 2(e)) and the negative control group (Figure 2(f)). There was no evidence of hyperemia or infiltration of mononuclear cells in the glomerular capillaries, dilatation of tubules, degeneration, or necrosis of the tubular epithelium compared to the control and negative control groups.

FIGURE 2Histopathological observation in liver and kidney tissues. All images were captured at a magnification of 20 ×. The scale bar is equal to 200 μm. The labeled structures include central vein (CV), hepatocyte (H), tubule (T), and glomerulus (G). (a) Liver of untreated mice, (b) liver of mice treated with 7% Tween 80 (negative control group), (c) liver of mice treated with 2000 mg/kg of *M. erythrophylla* extract, (d) liver of mice treated with 2000 mg/kg of *M. philippica* extract, (e) kidney of untreated mice, (f) kidney of mice treated with 7% Tween 80 (negative control group), (g) kidney of mice treated with 2000 mg/kg of *M. erythrophylla* extract, and (h) kidney of mice treated with 2000 mg/kg of *M. philippica* extract.

**Figure:**
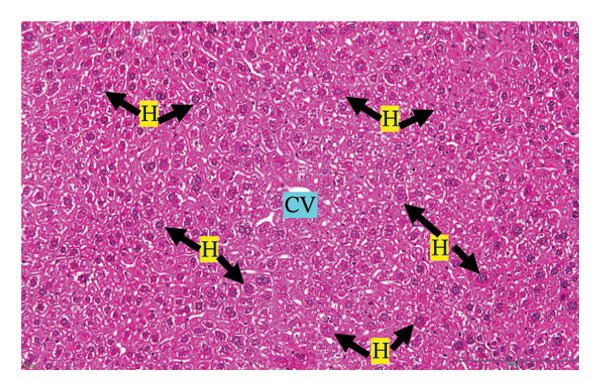
(a)

**Figure:**
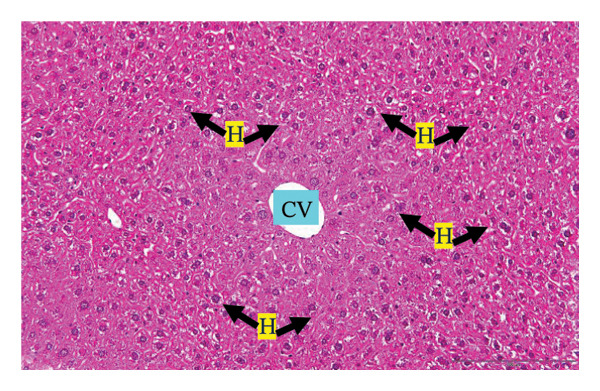
(b)

**Figure:**
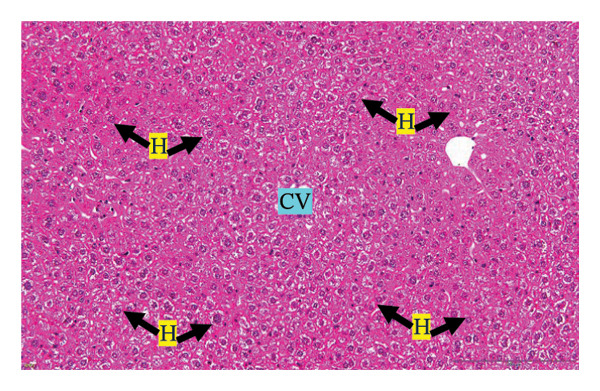
(c)

**Figure:**
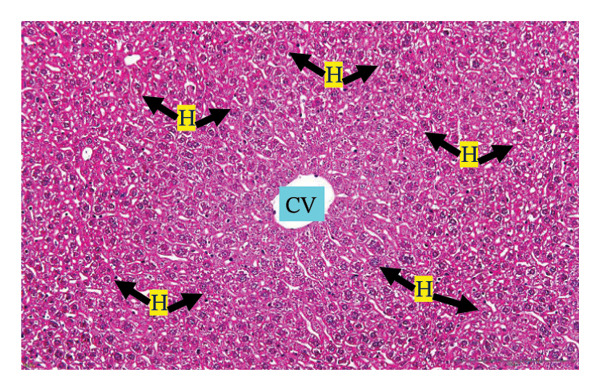
(d)

**Figure:**
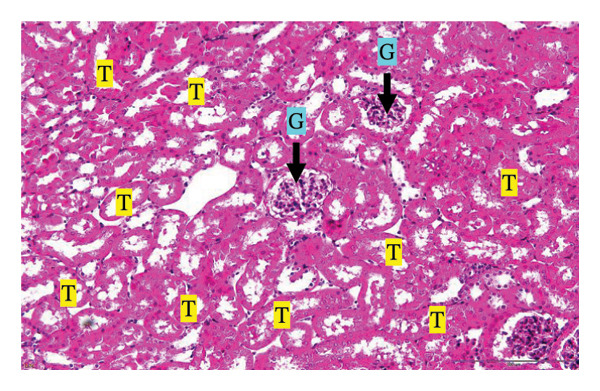
(e)

**Figure:**
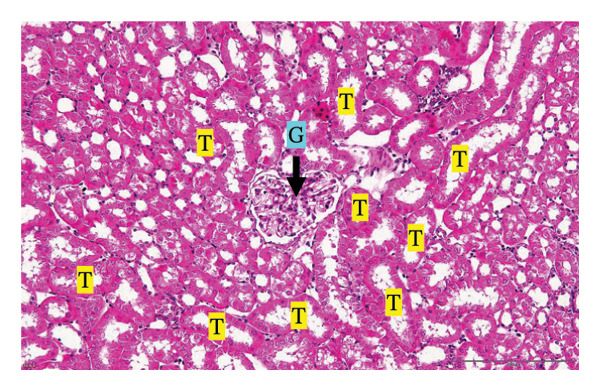
(f)

**Figure:**
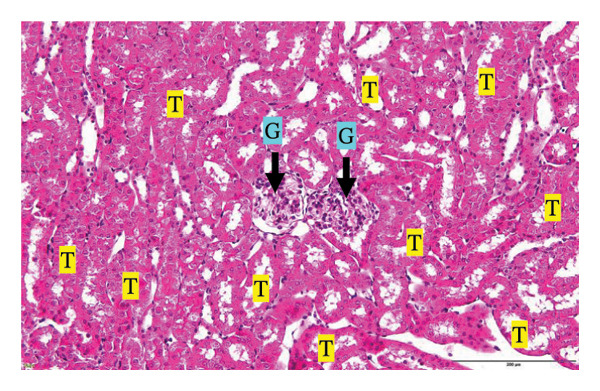
(g)

**Figure:**
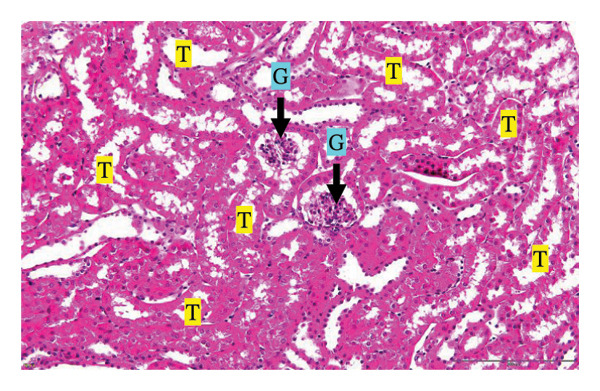
(h)

## 4. Discussion

Herbal medicines traditionally used in malaria‐endemic regions have contributed to the discovery of antimalarial drugs. Consequently, plants are regarded as promising and valuable sources of next‐generation antimalarial agents [36]. This study utilized an in vivo antimalarial model in rodents to consider the potential contribution of the immune system and the possible prodrug effect on the activity of plants against parasites [37].

Although previous studies have reported the in vitro antimalarial activities of *M. erythrophylla* and *M. philippica*, the present study provides novel in vivo evidence of their efficacy. The extracts demonstrated antimalarial activity against *P. berghei*‐infected mice, inhibiting parasite growth by 12.31%–71.02%. Notably, all extract doses, except for the 200 mg/kg dose of *M. erythrophylla*, exhibited significant parasitemia suppression, with *M. philippica* exhibiting a high suppression rate exceeding 70%. These findings indicate that the bioactive compounds in these plants may effectively inhibit *P. berghei* growth. Although their suppressive effects were lower than those of standard drugs, such as artesunate and CQ, the results highlight the potential of *M. erythrophylla* and *M. philippica* extracts as alternative or complementary antimalarial agents.

The observed antimalarial activities of the ethanolic leaf extracts of *M. erythrophylla* and *M. philippica* may be associated with their chemical constituents. In our previous phytochemical screening study, we found that the extracts contained flavonoids, terpenoids, and tannins. Further chemical profiling using GC‐MS, the plant extracts contained similar major chemical constituents, including phytol, β‐sitosterol, palmitic acid, and quercetin. Notably, phytol and β‐sitosterol correspond to terpenoids, and quercetin corresponds to flavonoids detected in the preliminary screening [27]. These results indicate that the GC‐MS findings are consistent with prior phytochemical analyses, providing additional evidence for the presence of bioactive compounds that may contribute to the antimalarial effects observed in vivo.

The biological activities of these compounds have been reported; the acyclic terpenoid phytol possesses antimalarial properties and was found to react with and block the plasmepsin enzyme in the malaria parasite [38, 39]. The β‐sitosterol molecule has antioxidant, immunomodulatory, and antimalarial characteristics. Its antimalarial action has been attributed to the inhibition of β‐hematin formation and the enzyme *P. falciparum* enoyl acyl‐carrier protein reductase (*pf*ENR) [40–43].

Palmitic acid is an immunomodulatory agent that stimulates pro‐inflammatory cytokine/neutrophil production [44]. The flavonoid quercetin has antiparasitic effects, including antimalarial properties, and its mechanisms have been linked to mitochondrial function disturbance and induction of nitric oxide and cytokine production [45, 46]. At a dose of 600 mg/kg, *M. philippica* demonstrated greater antimalarial efficacy than *M. erythrophylla*. This higher suppression may be due to the presence of unique bioactive compounds in *M. philippica*, such as 2‐pentadecanone, 6,10,14‐trimethyl‐cinnamic acid, and 7‐methyl‐Z‐tetradecen‐1‐ol acetate, which are not present in *M. erythrophylla* [27]. These compounds may act synergistically with shared constituents such as phytol, β‐sitosterol, palmitic acid, and quercetin, enhancing the overall antimalarial effect.

Toxicological evaluation of the extracts was performed to assess their safety in animals and potential applicability to humans [47]. No physical or behavioral abnormalities or mortality was observed during the acute toxicity investigation in mice orally administered the extracts. Therefore, the LD_50_ (lethal dose for 50% of the population) of *M. erythrophylla* and *M. philippica* was determined to be greater than 2000 mg/kg body weight.

The results of the food and water consumption analyses revealed that the extracts did not cause significant differences in values between the test and control groups. Thus, these findings indicate that the extracts did not affect the appetite of the mice. Alterations in body weight serve as indicators of overall health [48]. The findings of this study indicated that changes in body weight did not exhibit a statistically significant difference among the groups. This finding implies that *M. erythrophylla* and *M. philippica* did not produce negative effects. For biochemical evaluation, only AST levels in mice treated with the extract were significantly elevated compared to those in the control groups. However, AST levels remained within the standard reference range for normal mice (24–472 U/L) [35].

This pattern may reflect a mild physiological response to xenobiotic metabolism rather than true hepatotoxicity. Transient increases in AST have been documented to be associated with metabolic adaptation, increased hepatic enzyme activity, and mild cellular stress, without corresponding structural liver damage [49]. Moreover, isolated AST elevation is less indicative of hepatocellular injury than is ALT elevation, which is considered a more specific marker of liver damage [50]. The absence of concurrent ALT elevation, together with normal histopathological findings, further supports the interpretation that the observed increase in AST levels is more likely related to mild physiological or metabolic stress rather than to early signs of toxicity. Nonetheless, monitoring AST trends in future studies, particularly at higher doses or with prolonged exposure, would be valuable to confirm the safety profile of the extract.

Furthermore, histopathological examination of the liver tissue revealed no significant abnormalities, with a normal liver architecture. This correlation between the biochemical and histopathological findings implies that transient AST elevation may reflect mild physiological adaptation rather than overt toxicity. AST is expressed in multiple organs, including the cardiac and skeletal muscles, kidneys, brain, and pancreas [50]. Therefore, the observed increase in AST may not reflect hepatic toxicity and may be related to nonhepatic stress or adaptive metabolic responses to the extract. Importantly, no histopathological changes were detected in the liver tissue, supporting the overall safety of the extracts at the tested doses.

Elevated AST levels indicate tissue damage due to conditions such as liver inflammation, septic shock, myocardial infarction, and muscle injury. Nonhepatic causes included skeletal muscle damage (54.2%), cardiac muscle damage (39.1%), and hematological disorders (6.7%) [51]. AST was the first cardiac biomarker used to diagnose acute myocardial infarction; however, its use has declined due to its lack of cardiac specificity. In patients with extensive myocardial injury, AST levels are often elevated, which is commonly observed in patients with acute coronary syndrome. Elevated AST in cardiac damage stems from myocardial release during stress, ischemia, necrosis, and reperfusion injury [51, 52].

The extracts did not affect the kidneys, as indicated by normal histology findings and the lack of a significant difference of BUN and creatinine levels among all groups. This study demonstrated that *M. erythrophylla* and *M. philippica* extracts did not directly affect the liver or kidneys. This study was primarily concerned with determining the toxicity of the extracts to the liver and kidneys, as these organs are susceptible to harm from oral metabolism [53].

Although a single‐dose limit test at 2000 mg/kg was used, which is below the OECD Test Guideline 425 maximum of 5000 mg/kg, this dose is consistent with the OECD recommendations for preliminary acute toxicity testing when no prior evidence of extreme toxicity exists and is sufficient to provide meaningful initial safety data. However, future studies should explore higher doses and investigate the potential effects on other organs, including the cardiac and skeletal muscles, brain, and pancreas, to further confirm the overall safety and support the evaluation of their antimalarial activity. Additionally, the antimalarial properties of the active compounds as well as the mechanisms of action of *M. erythrophylla* and *M. philippica* should be further evaluated.

Although the selection was based on IC_50_ values from in vitro assays, in vivo outcomes may be influenced by factors such as host immune responses, pharmacokinetic and pharmacodynamic interactions, and the presence of prodrugs. Additionally, only male mice were used in this study, which may not account for potential sex‐based differences in drug responses. This limitation, along with the potential efficacy of aqueous extracts, will be addressed in future studies. Future studies should include larger and more diverse animal populations, longer‐term toxicity assessments, and pharmacokinetic evaluations to comprehensively assess the safety and antimalarial efficacy of *M. erythrophylla* and *M. philippica* extracts.

## 5. Conclusions

*M. erythrophylla* and *M. philippica* leaf extracts demonstrated significant dose‐dependent antimalarial activity in vivo and were safe at up to 2000 mg/kg, with only mild and nonpathological AST elevation. These findings support further isolation of active compounds and long‐term safety studies before considering translational applications.

## Author Contributions

P.C., A.P., W.P., A.K., and C.P. designed the study. P.C., A.P., W.P., A.K., P.V., and C.P. performed the experiments. P.C., A.P., W.P., A.K., P.V., A.W.S., and C.P. analyzed the data. P.C., A.W.S., and C.P. reviewed the statistical analysis. P.C., A.P., W.P., and C.P. drafted the manuscript. P.V. and A.W.S. reviewed and edited the manuscript.

## Funding

This work was supported by the Walailak University Plant Genetic Conservation Project under the Royal Initiation of Her Royal Highness, Princess Maha Chakri Sirindhorn, RSPG‐WU‐33/2566, Walailak University, under the international research collaboration scheme.

## Disclosure

All the authors have read and approved the final version of the manuscript.

## Conflicts of Interest

The authors declare no conflicts of interest.

## Data Availability

The data associated with this study are included in this article. Additional files are available from the corresponding author upon request.
